# Supplementary material for: The interactome of intact mitochondria by cross-linking mass spectrometry provides evidence for coexisting respiratory supercomplexes
Source: Mol Cell Proteomics. 2017 Dec 8;17(2):216–32. doi: 10.1074/mcp.RA117.000470 (PMC5795388; doi:10.1074/mcp.RA117.000470)
Supplement: Supplemental Data [file supp_RA117.000470_133922_0_supp_23978_fzffwf.zip › spectra_annotation/mito_spectra_annotation/263-1-16-1-6-1.pdf]

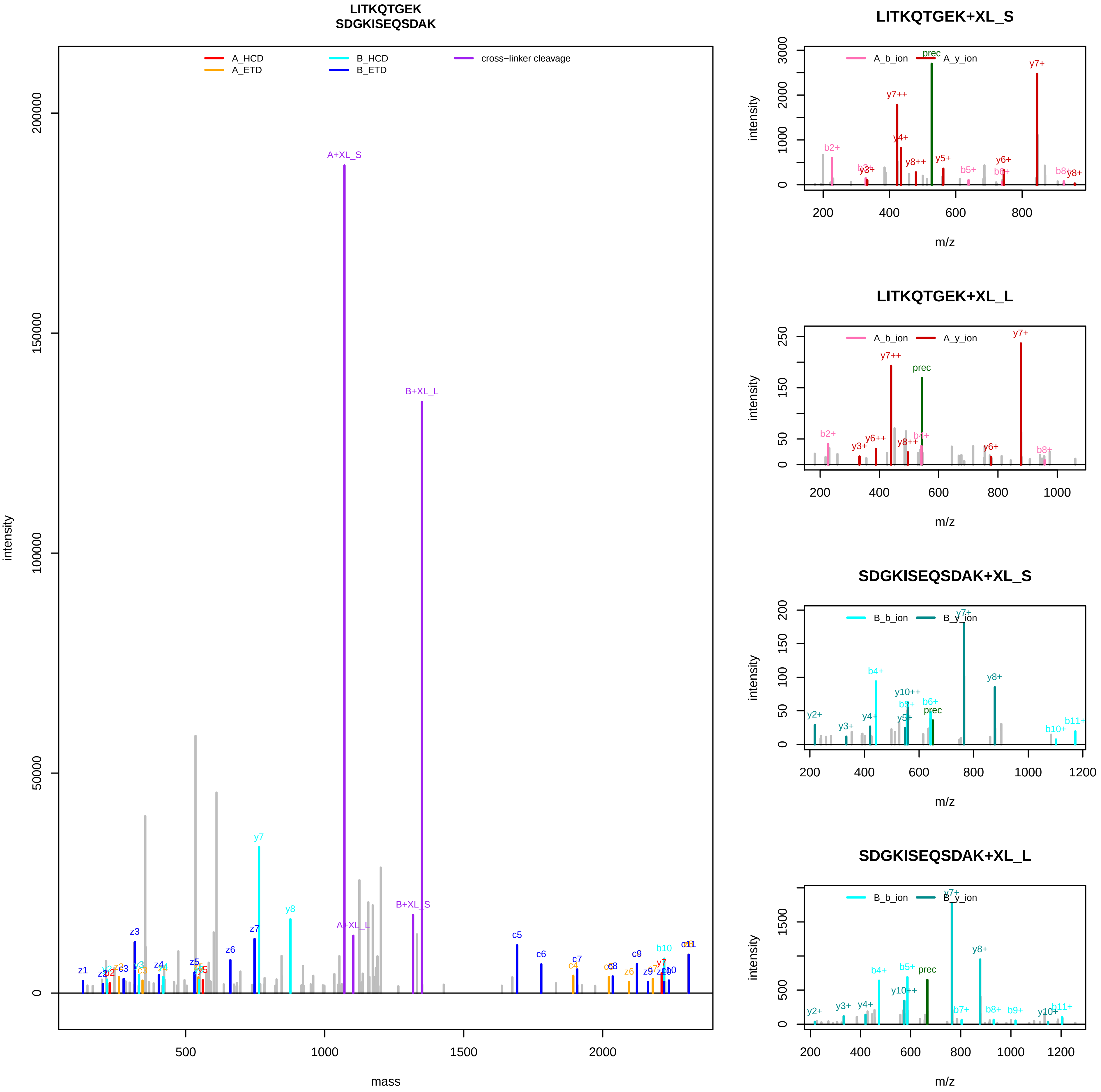

LITKQTGEK
SDGKISEQSDAK
A_HCD
A_ETD
B_HCD
B_ETD
cross−linker cleavage
A+XL_S
B+XL_L
A+XL_L
B+XL_S
y7
y8
z1
z3
z4
z5
z6
z7
z9
c3
c5
c6
c7
c8
c9
c11
b10
intensity
mass
0
50000
100000
150000
200000
500
1000
1500
2000
LITKQTGEK+XL_S
A_b_ion
A_y_ion
prec
y7+
y7++
y4+
y8++
y5+
b2+
b5+
y6+
y8+
y3+
b8+
intensity
m/z
0
1000
2000
3000
200
400
600
800
LITKQTGEK+XL_L
A_b_ion
A_y_ion
y7+
y7++
prec
b2+
y3+
y6++
y8++
b4+
y6+
b8+
intensity
m/z
0
50
150
250
200
400
600
800
1000
SDGKISEQSDAK+XL_S
B_b_ion
B_y_ion
y7+
b4+
y8+
y10++
b5+
b6+
prec
y2+
y3+
y4+
y5+
b10+
b11+
intensity
m/z
0
50
100
150
200
200
400
600
800
1000
1200
SDGKISEQSDAK+XL_L
B_b_ion
B_y_ion
y7+
y8+
b4+
b5+
prec
y10++
y2+
y3+
y4+
b7+
b8+
b9+
y10+
b11+
intensity
m/z
0
500
1500
200
400
600
800
1000
1200
